# Olanzapine versus low-dose dexmedetomidine for delirium in patients with critical illness: a randomized clinical trial

**DOI:** 10.1093/ajrccm/aamag235

**Published:** 2026-05-12

**Authors:** Jinjie Liu, Chao Wang, Ximing Nie, Rongli Yang, Mengxing Wang, Xiao Ma, Kai Xu, Guozhi Wu, Di Li, Sibo Liu

**Affiliations:** Department of Neurology, Central Hospital of Dalian University of Technology, Dalian, China; Faculty of Medicine, Dalian University of Technology, Dalian, China; Intensive Care Unit, Central Hospital of Dalian University of Technology, Dalian, China; Department of Neurology, Neurocritical Care Unit, Beijing Tiantan Hospital, Capital Medical University, Beijing, China; China National Clinical Research Center for Neurological Diseases, Beijing, China; Faculty of Medicine, Dalian University of Technology, Dalian, China; Intensive Care Unit, Central Hospital of Dalian University of Technology, Dalian, China; China National Clinical Research Center for Neurological Diseases, Beijing, China; Department of General Medicine, Central Hospital of Dalian University of Technology, Dalian, China; Department of Neurosurgery, Central Hospital of Dalian University of Technology, Dalian, China; Intensive Care Unit, Central Hospital of Dalian University of Technology, Dalian, China; Faculty of Medicine, Dalian University of Technology, Dalian, China; Department of Neurological Intervention and Intensive Care, Central Hospital of Dalian University of Technology, Dalian, China; Faculty of Medicine, Dalian University of Technology, Dalian, China; Intensive Care Unit, Central Hospital of Dalian University of Technology, Dalian, China

*To the Editor:*

Delirium affects approximately 40% of critically ill patients and is associated with prolonged ventilation and increased mortality.^1^^,^^2^ While current guidelines recommend the use of dexmedetomidine over propofol for light sedation in ventilated patients admitted to the intensive care unit (ICU), its efficacy for treating established delirium remains unclear despite evidence that it reduces delirium incidence.^2^ Conversely, antipsychotics are used to treat delirium, although there is insufficient evidence to support their efficacy.^2-4^ According to our prior observations, olanzapine may be a potential safe antipsychotic in critically ill patients.^5^ Therefore, we conducted a randomized controlled trial (RCT) to compare the efficacy and safety of olanzapine and dexmedetomidine in ICU patients with established delirium.

## Methods

This single-center, tertiary referral-based RCT was approved by the ethics committee of Central Hospital of Dalian University of Technology (20201-045-01) and registered prospectively (ChiCTR2100049820). Adult patients (aged ≥18 years) admitted to the ICU were screened for delirium daily using the Confusion Assessment Method for the Intensive Care Unit (CAM-ICU).^6^ Patients diagnosed with delirium were eligible if they could be enrolled within 24 hours.^3^ Patients with neuroleptic malignant syndrome, drug allergies, metastatic cancer, pregnancy/breastfeeding, alcohol withdrawal, history of torsades de pointes, QTc >440 ms, planned renal replacement therapy, current antipsychotic use, conditions preventing reliable evaluations (such as incarceration), or participation in another trial were excluded.

Patients were randomized 1:1 to receive oral olanzapine (2.5-20 mg/day) plus intravenous placebo or intravenous low-dose dexmedetomidine (0.1-0.7 μg/kg/hour)^7^ plus oral placebo. The olanzapine dosage was increased by 5 mg/day in CAM-ICU–positive patients and decreased by 2.5 mg/day in patients with 2 consecutive CAM-ICU–negative days. Dexmedetomidine was adjusted by 0.1 μg/kg/hour every 15 minutes to maintain a target Richmond Agitation and Sedation Scale score of −2 to 1 (increase if >1; decrease if < −2), paused daily at 7:00 am for CAM-ICU assessment, and halved if negative.

Study drugs were discontinued after 2 consecutive CAM-ICU–negative days, 7 days, ICU discharge, death, or for safety, whichever occurred first.^3^ If agitation persisted at the maximum drug dose, concomitant analgesic or sedative use was permitted.^7^ Drugs were restarted at the last dose if a new delirium episode arose.

The primary efficacy outcome was the number of days alive without delirium/coma during 7-day intervention (or until ICU discharge/death, whichever occurred first) from randomization.^3^ Secondary efficacy outcomes included delirium duration, intubation rate and ICU stay postrandomization, delirium recurrence, drug combination, and mortality rate (at discharge, 30 days, and 90 days). Safety outcomes included hypotension (blood pressure <80/50 mm Hg), bradycardia (heart rate <40 beats per minute), sinus arrest, respiratory depression, extrapyramidal reactions (using a modified Simpson–Angus Scale), torsades de pointes, and antipsychotic malignant syndrome.

All data were analyzed using an intention-to-treat approach (SAS version 9.1). The primary outcome was analyzed using an unadjusted proportional-odds logistic regression model. A multivariate Cox regression model was used to analyze 90-day mortality. Other secondary outcomes were analyzed using a multivariate generalized linear or logistic regression model. Models were adjusted for age, baseline Acute Physiology and Chronic Health Evaluation (APACHE) II score, ventilation and/or tracheal intubation, days of ICU stay before randomization, and drug combination, all selected post hoc. A 2-sided *P* value <.05 indicated statistical significance.

## Results

Between 2021 and 2025, 918 of 2462 patients developed delirium; 313 of 918 could not be enrolled within 24 hours, leaving 605 eligible. Of these patients, 348 met the exclusion criteria and 69 declined to participate. Ultimately, 188 underwent randomization and all accepted intended drugs. Crossover between groups was documented for 20 patients (10 per group). Patient characteristics are shown in Table 1.

**Table 1:** Baseline characteristics of the study participants, by treatment group.

Characteristics	**Total (*N*** = **188)**	**Olanzapine (*n*** = **94)**	**Dexmedetomidine (*n*** = **94)**
**Age, y, mean (SD)**	70 (15)	70 (14)	71 (16)
**Sex, male, No. (%)**	121 (64.4)	61 (64.9)	60 (63.8)
**Height, cm, median (IQR)**	170 (165-175)	170 (165-175)	170 (164-175)
**Weight, kg, median (IQR)**	70 (60-78)	70 (62-75)	67 (60-78)
**Medical history, No. (%)**			
** Hypertension**	98 (52.1)	50 (53.2)	48 (51.1)
** Diabetes mellitus**	57 (30.3)	33 (35.1)	24 (25.5)
** Atrial fibrillation**	11 (5.9)	4 (4.3)	7 (7.4)
** Hyperlipidemia**	3 (1.6)	1 (1.1)	2 (2.1)
** Cardiovascular disease**	20 (10.6)	12 (12.8)	8 (8.5)
** Previous stroke**	16 (8.5)	10 (10.6)	6 (6.4)
** Current smoking**	34 (18.1)	11 (11.7)	23 (24.5)
** Alcohol**	27 (14.4)	9 (9.6)	18 (19.2)
** Drug abuse**	1 (0.5)	1 (1.1)	0 (0.0)
** Anxiety or depression**	1 (0.5)	1 (1.1)	0 (0.0)
** Respiratory failure**	31 (16.5)	13 (13.8)	18 (19.1)
** Liver disease**	3 (1.6)	1 (1.1)	2 (2.1)
** Heart failure**	21 (11.2)	11 (11.7)	10 (10.6)
** Tumor**	1 (0.5)	1 (1.1)	0 (0.0)
**Diagnosis at ICU admission, No. (%)**			
** Sepsis or ARDS**	20 (10.6)	11 (11.7)	9 (9.6)
** Nontraumatic major surgery**	25 (13.3)	9 (9.6)	16 (17.0)
** Respiratory failure**	31 (16.5)	13 (13.8)	18 (19.1)
** Renal failure**	6 (3.2)	4 (4.3)	2 (2.1)
** Liver failure**	3 (1.6)	1 (1.1)	2 (2.1)
** Gastrointestinal diseases**	28 (14.9)	11 (11.7)	17 (18.1)
** Acute coronary syndrome**	11 (5.9)	8 (8.5)	3 (3.2)
** Heart failure**	21 (11.2)	11 (11.7)	10 (10.6)
** Other^a^**	43 (22.9)	26 (27.7)	17 (18.1)
**Disease severity before randomization, median (IQR)**			
** APACHE II score^b^**	16 (13-19)	16 (12-19)	17 (14-19)
** GCS score^b^**	12 (11-14)	13 (11-14)	12 (12-14)
** DRS-R-98 score**	25 (21-28)	25 (21-28)	25 (22-27)
** Time from ICU admission to randomization, d**	2 (1-3)	3 (1-4)	2 (1-3)
** Ventilation and/or tracheal intubation, No. (%)**	76 (40.4)	33 (35.1)	43 (45.7)
**Vital signs at ICU admission^b^**			
** Heart rate, beats/min, median (IQR)**	89 (78-104)	88 (78-98)	92 (80-110)
** Respiratory rate, breaths/min, median (IQR)**	18 (16-20)	18 (17-20)	18 (16-21)
** Systolic BP, mm Hg, mean (SD)**	124 (23)	126 (22)	122 (23)
** Diastolic BP, mm Hg, mean (SD)**	70 (14)	72 (12)	68 (16)
**Type of delirium**			
** Hyperactive**	47 (25.0)	21 (22.3)	26 (27.6)
** Mixed**	109 (58.0)	59 (62.8)	50 (53.2)

Abbreviations: APACHE, Acute Physiology and Chronic Health Evaluation; ARDS, acute respiratory distress syndrome; BP, blood pressure; DRS-R-98, Delirium Rating Scale–Revised-98; GCS, Glasgow Coma Scale; ICU, intensive care unit; IQR, interquartile range; SD, standard deviation.

a Other diagnoses at ICU admission included multiple organ dysfunction syndrome, shock, severe acid-base imbalances, severe hyperosmolar syndrome, malignant arrhythmias, intractable hyperthermia, pulmonary embolism, etc.

b APACHE II score, GCS score, and vital signs were recorded using worst values over previous the 24 hours from the time of study enrollment.

**Figure 1:**
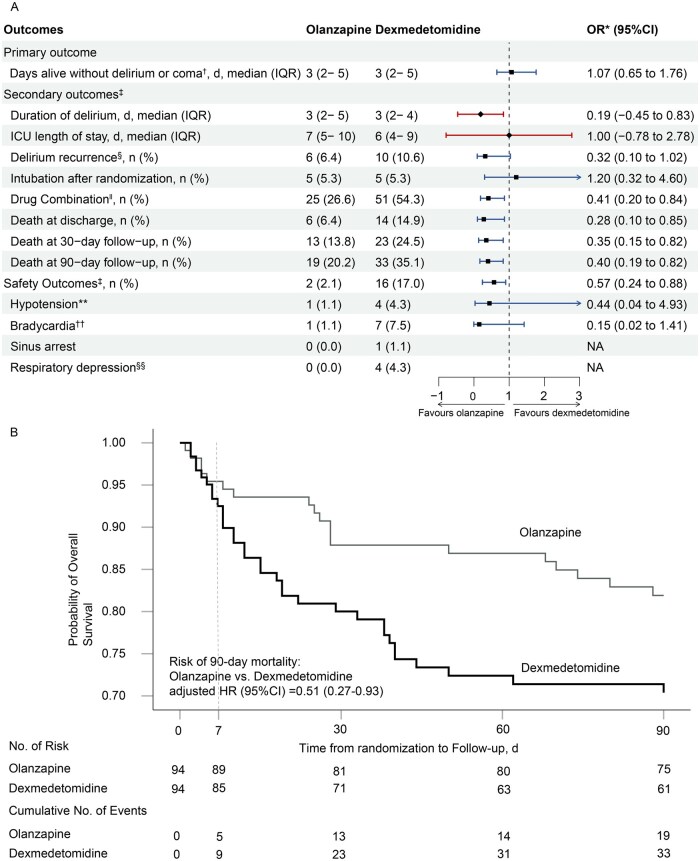
Outcomes. (A) Patients’ outcomes in the olanzapine and dexmedetomidine groups. (B) Cox proportional hazards model analysis of 90-day mortality postrandomization. *Effect size and CIs were reported using common ORs for ordinal outcomes (blue), ORs for categorical outcomes (blue), and β values for continuous outcomes (red). ^†^Analysis was adjusted for potential confounders, including age, baseline APACHE II score, ventilation and/or tracheal intubation, days of ICU stay before randomization, and drug combination. ^‡^Defined as the number of days alive without delirium/coma during the 7-day intervention period. ^§^Defined as any new episode of delirium occurring between the discontinuation of treatment and the 90-day follow-up. ^‖^Defined as the use of concomitant analgesics or sedatives beyond the assigned study drug to manage agitation. ^**^Defined as blood pressure < 80/50 mm Hg related with intervention. ^††^Defined as heart rate <40 beats/min related with intervention. ^§§^Defined as respiratory rate <10 breaths/min or oxygen saturation <90% related with intervention. Other severe adverse effects including extrapyramidal reaction, torsade de pointes, and antipsychotic malignant syndrome were not observed. Abbreviations: APACHE, Acute Physiology and Chronic Health Evaluation; CI, confidence interval; HR, hazard ratio; ICU, intensive care unit; interquartile range; NA, not applicable; OR, odds ratio.

The median number of days alive without delirium/coma was 3 (IQR, 2-5) in both groups, with no significant difference (odds ratio, 1.07 [95% CI, 0.65-1.76]) (Figure 1A). The mortality rates in the olanzapine group and dexmedetomidine group were 6.4% vs 14.9% at discharge, 13.8% vs 24.5% at 30 days, and 20.2% vs 35.1% at 90 days. In the prespecified exploratory endpoints of 90-day mortality, the dexmedetomidine group exhibited a higher rate than the olanzapine group (adjusted hazard ratio, 0.40 [95% CI, 0.19-0.82]; e-value, point estimate 4.30 [CI bound: 1.53]) (Figure 1B). The total adverse event rate was statistically higher with dexmedetomidine (17.0% vs 2.1%), predominantly hypotension, bradycardia, and respiratory depression (Figure 1A). Other secondary outcomes were comparable between groups. Sensitivity analysis excluding cross-over patients or adjusting for different covariates yielded similar results.

## Discussion

We found no statistically significant difference in the primary outcome of number of days alive without delirium/coma, with a wide CI precluding a definitive conclusion of equivalent efficacy between the groups.

The prespecified exploratory analysis revealed higher 90-day mortality in the dexmedetomidine group. However, the trial was not powered to detect difference in mortality and is therefore at increased risk of producing false-positive results. Moreover, a numerical difference in baseline mechanical ventilation rates and greater adjunct sedative use in the dexmedetomidine group confounded comparison despite adjustment. The observed difference yielded an e-value of 4.30, suggesting moderate robustness to unmeasured confounding. Notably, the postdischarge mortality rate was higher than previously reported and expected.^8^^,^^9^ Furthermore, the survival curves separate at approximately 7 days postrandomization, complicating causal interpretation, as this could reflect either a late effect of the sedative strategy or the underlying disease trajectory.

As observed, dexmedetomidine was associated with more adverse events in our study, primarily hypotension and bradycardia, consistent with previous studies.^10^ The low-dose dexmedetomidine protocol, aimed at preserving alertness,^7^ likely contributed to the high rate of adjunct sedative use, which may be partly related to the increased incidence of respiratory depression, an uncommon adverse event of dexmedetomidine.

Our study is limited by its single-center design and potential selection bias, affecting its generalizability. The wide CI for the primary outcome precludes definitive conclusions on equivalence. The exploratory mortality difference remains susceptible to confounding from differential concomitant sedation and baseline bias despite multivariate adjustment. Furthermore, the separation of survival curves after day 7 complicates interpretation.

In summary, this trial revealed no difference in the number of days alive without delirium/coma. The observed difference in mortality is hypothesis-generating and requires further validation.

## Supplementary Material

aamag235_Supplementary_Data

## Data Availability

Requests should be directed to the corresponding author.
